# Genome-wide analysis of purple acid phosphatase structure and expression in ten vegetable species

**DOI:** 10.1186/s12864-018-5022-1

**Published:** 2018-08-31

**Authors:** Lulu Xie, Qingmao Shang

**Affiliations:** Key Laboratory of Biology and Genetic Improvement of Horticultural Crops of Ministry of Agriculture, Institute of Vegetables and Flowers, Chinese Academy of Agricultural Sciences, Beijing, 100081 China

**Keywords:** Phosphorous, PAP enzymes, Protein structure, Gene expression, Phosphate use efficiency

## Abstract

**Background:**

Acquisition of external phosphorus (P) and optimisation of internal P are essential for plant growth and development, and insufficient availability of P in soils is a major challenge in agriculture. Members of the purple acid phosphatase (PAP) family of enzymes are candidates for increasing P use efficiency. Herein, we identified PAP homologs in the genomes of 10 vegetable species, along with *Arabidopsis thaliana* and *Amborella trichopoda* as references, to provide fundamental knowledge for this family.

**Results:**

Phylogenetic analysis of protein sequences revealed nine distinct clades, indicating that functional differentiation of extant PAPs was established prior to the emergence of early angiosperms, and conserved among homologs in each clade. Analysis of transcript abundance in different tissues (root, stem, leaf, flower, and fruit) and following phosphates (Pi) starvation treatments from published RNA-seq transcriptome datasets facilitated comprehensive evaluation of expression patterns, and some groups of tissue-specific and Pi starvation-induced PAPs were characterised. Conserved motifs identified from upstream sequences of homologs that are highly expressed in particular tissues or following starvation treatment suggests that divergence in *PAP* gene expression is associated with *cis*-acting elements in promoters.

**Conclusions:**

The genome-wide analysis of PAP enzyme structure and transcriptional expression patterns advance our understanding of PAP family in vegetables genomes. Therefore, PAP homologs with known enzyme structures and expression profiles could serve as targets for plant breeding and/or genetic engineering programs to improve P acquisition and use.

**Electronic supplementary material:**

The online version of this article (10.1186/s12864-018-5022-1) contains supplementary material, which is available to authorized users.

## Background

Phosphorus (P) is a fundamental constituent of nucleic acids and phospholipids, and key components in energy metabolism, signal transduction and enzymatic reactions. Phosphorus use is a limiting factor for plant growth and development in most soils. The primary forms taken up by roots are orthophosphates (Pi mainly in the form of H_2_PO_4_^−^ or HPO_4_^2−^), which rarely exceed 10 μM in soil water, even in the more fertile soils [1]. In agricultural practices, phosphate fertilisers are routinely applied, but a significant amount will coprecipitate and become unavailable for plants [2], and the final use efficiency is estimated to be only 20% [3]. Due to rapidly diminishing phosphorus stocks, and environmental problems such as surface water eutrophication, excessive application of chemical fertilisers is unsustainable.

The concept of P use efficiency is now widely accepted [4, 5]. In soils, organic phosphorus (Po) in the form of monoesters or diesters generally accounts for ~ 30−80% of the total P content [6]. In plants, Pi or P-esters in vacuolar and senescing tissues constitute buffering pools [7]. Therefore, more active and appropriate mobilisation of organic P sources, including maximising the efficiency of external P acquisition and reprioritising internal P use, has the capacity to enhance overall P use efficiency.

The release of Pi from esters is the key step in Po utilisation. Acid phosphatases (EC: 3.1.3.2) catalyse the hydrolysis of Pi from a broad range of phosphomonoesters and act optimally at acidic pH [8]. Phosphatases tend to be nonspecific, or the substrate specificity may be defined but not strict, and enzymes of the latter type may play a specific metabolic role [9, 10]. Based on function, acid phosphatases can be divided into secreted acid phosphatases (SAPs) and intracellular acid phosphatase (IAPs). Most IAPs are present in the vacuole as soluble proteins, while SAPs are localised in the cell wall or secreted into the rhizosphere [9]. Some acid phosphatases such as AtPAP26 appear to possess both SAP and IAP functions [10, 11]. All known SAP and IAP isozymes belong to the purple acid phosphatase (PAP) family [12, 13].

PAPs are widespread among plants, mammals and microbes [14]. Three-dimensional structures of several PAPs have been determined, including those from red kidney bean (GenBank: P80366, PDB: 1KBP) [15] and pig (GenBank: P09889, PDB: 1UTE) [16]. All known PAPs have two closely-spaced metal ions forming a dinuclear center that carries out hydrolytic reactions, and their distinctive purple color is due to a charge transfer transition from a conserved tyrosine ligand to a ferric ion in the active site [17]. In plants, the dinuclear metal centres are generally Fe(III)-Zn(II) or Fe(III)-Mn(II), as is the case in soybean, sweet potato and red kidney bean [18–20]. Seven amino acid residues (shown in bold font) embedded in five conserved blocks, **D**xG-x(n)-G**D**x(2)**Y**-x(n)-G**N**H[ED]-x(n)-Vx(2)**H**-x(n)-G**H**x**H** (where x indicates any amino acid), were identified invariant among PAPs from different sources [14]. These conserved sequences facilitated the genome-wide identification of a large number of *PAP* genes in *Arabidopsis thaliana*, *Oryza sativa*, *Glycine max* and *Zea mays* [21–24]. According to previous studies, PAPs are traditionally classified into two groups based on molecular weight and the mode of interaction; ~ 35 kDa form are monomeric, and ~ 55 kDa form are homodimeric [17]. Homology modeling shows that the smaller type lack the NH_2_-domain, which does not perform a catalytic function, which is present in larger PAPs [14]. Furthermore, heterodimeric PAPs (63 kDa and 57 kDa) and larger PAPs (84 kDa) have been reported in tomato, implying more complicated diversification [12, 13].

*PAP* genes are expressed throughout all plant tissues, but tissue-specific expression patterns are evident. Early research on lupin (*Lupinus albus*) showed that a secreted APase is expressed at higher levels in roots than shoots [25]. In tomato (*Solanum lycopersicum*), protein levels of several selected PAPs are different in leaf, stem and root tissues [26]. Research on *Arabidopsis* revealed that some *PAPs* are expressed at moderate levels in roots, stems, leaves, flowers and siliques, while others are specifically expressed in particular tissues such as flowers [27]. In other work, AtPAP10 in *Arabidopsis* was shown to be predominantly associated with the root surface [28]. In addition to their tissue-specific properties, *PAP* genes are usually regulated by Pi levels in the external environment, and they play important roles in Pi starvation-inducible responses. Early studies on nine crop species demonstrated the dramatic secretion of APs by roots [29], and both SAPs and IAPs were later found to be highly expressed after Pi starvation for several days [26]. Approximately half of all PAPs in *Arabidopsis* and soybean are upregulated under phosphate starvation conditions, even with different degrees [21, 23, 30, 31].

Enzymes such as AtPAP10, AtPAP12 and AtPAP26 with dominant functions account for most of the SAP activity [31, 32]. Regarding transcriptional regulation, *trans-*factors PHR1 (MYB), WRKY75, bHLH32 and ZAT6 (C2H2 zinc finger-type) were proposed to control the expression of PAPs in Arabidopsis [33]. However, studies on orthologues in vegetable genomes are only in their early stages [34]. Due to the complexity of developmental and environmental regulation, *cis*-element combinations in promoter region are highly complex [35]. Details of conserved regulatory regions in members of the PAP family, especially in other species, are limited.

PAP enzyme family members are excellent candidates for genetic engineering and intensive cultivation to enhance P efficiency, which would be of great agricultural and ecological benefit. However, fundamental knowledge in this area is clearly needed, since systemic research of this enzyme family focusing on vegetable crops has not been reported, despite the excessive use of phosphate fertilisers in soils used for vegetable cultivation. Herein, we performed a comprehensive analysis of 10 domesticated plant species from Cucurbitaceae, Solanaceae and Brassicaceae to investigate the structures and gene expression patterns of PAP enzymes.

## Results

### PAP copy number variation in vegetable genomes

**Fig. 1:**
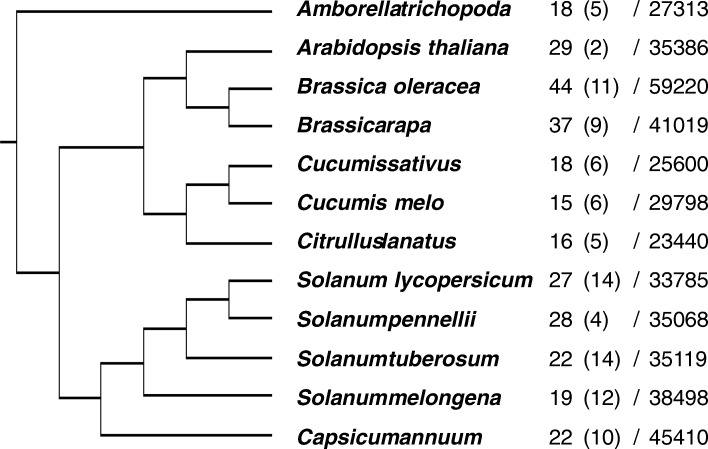
Phylogenetic relationships between the chosen genomes. Numbers of purple acid phosphatase (PAP) genes and total genes are followed by species names. Numbers in brackets indicate PAP hits with truncated metal ion binding regions

We chose 12 plant species for which whole-genome sequencing has been accomplished, including three Brassicaceae (*Arabidopsis thaliana*, *Brassica oleracea* and *B. rapa*), five Solanaceae (*Solanum lycopersicum*, *S. pennellii*, *S. tuberosum*, *S. melongena* and *Capsicum annuum*), three Cucurbitaceae (*Cucumis sativus*, *C. melo* and *Citrullus lanatus*), and one basal angiosperm (*Amborella trichopoda*; Fig. 1). Nine of these are vegetable species widely cultivated across the world. Genes putatively encoding complete conservative catalytic regions were identified (see Methods).

After searching target sequences, the copy number of PAP family members in these genomes was determined. Whole-genome ploidisation has caused multiple increases in the number of chromosomes and genes during evolution, and even where chromosomes and genes have been lost, the total number of genes has continually increased. Extra copies of some genes can provide opportunities to evolve additional functions via dosage effects, which can help new species to adapt to external environments. If genes of a given group are favourable for fitness, more copies are likely to be retained. The copy number of PAP family genes does not appear to have risen or fallen dramatically during the evolution of these plants; rather, the copy number has remained relatively stable and PAP genes account for a small proportion of all genes (Fig. 1). As occurred in Brassicaceae, after speciation from a common ancestor of *Arabidopsis* and *Brassica*, the ancestor of *B. rapa* and *B. oleracea* experienced a triploidisation event [36]. The increase in copy number of complete PAP genes in *B. rapa* (37) and *B. oleracea* (44) compared with *A. thaliana* (29) is generally equal to the increase in putative total genes (41,019, 59,220 and 35,386). In Solanaceae, the ancestor of *S. tuberosum* experienced a diploidisation event that did not occur in the ancestor of *S. lycopersicum* [37], but the copy number in *S. tuberosum* (22) is less than that of *S. lycopersicum* (27). In Cucurbitaceae, the common ancestor of the three species experienced a tetraploidy event [38], and the copy number in *C. sativus, C. melo* and *C. lanatus* has been maintained at 18, 15 and 16, respectively, compared with total gene numbers of 25,600, 29,798 and 23,440. Also, homologous copies found in CDS sequences but with truncated conserved regions were listed as pseudo-genes (Fig. 1, numbers in brackets). Similar to complete copies, the number of incomplete copies also remained stable. There was a slight difference in the proportion of pseudo-genes between families (~ 25% in the three species of Cucurbitaceae, and ~ 35% in the five species of Solanaceae.) This may suggest that PAP genes have not experienced a strong selection bias, at least in the plants chosen in this study.

### Phylogenetic relationships and divergence in gene structures and selection pressure of PAP homologs

**Fig. 2:**
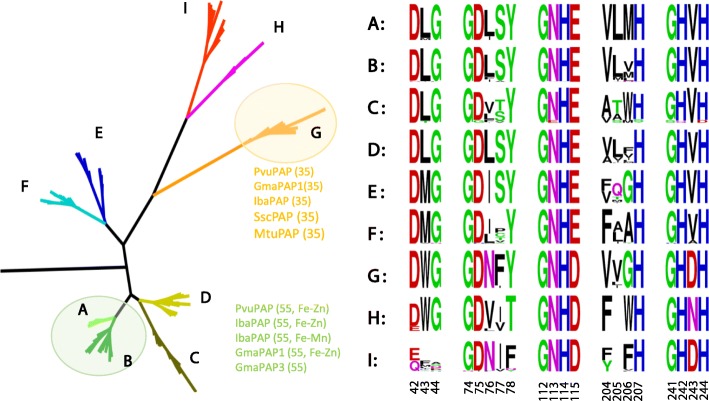
Phylogenetic relationship and active site regions of PAP amino acid sequences. Left: Simplified Maximum likelihood (ML) tree of 296 PAP amino acid sequences. The items in green are functional characterised reference sequences with the highest similarity to clade A and B and the items in yellow are reference sequences with the highest similarity to clade G [17]. Right: the corresponding sequences of five conserved active site regions (numbers below refer to *Phaseolus vulgaris* AF236109). Displayed by WebLogo: http://weblogo.berkeley.edu/

**Fig. 3:**
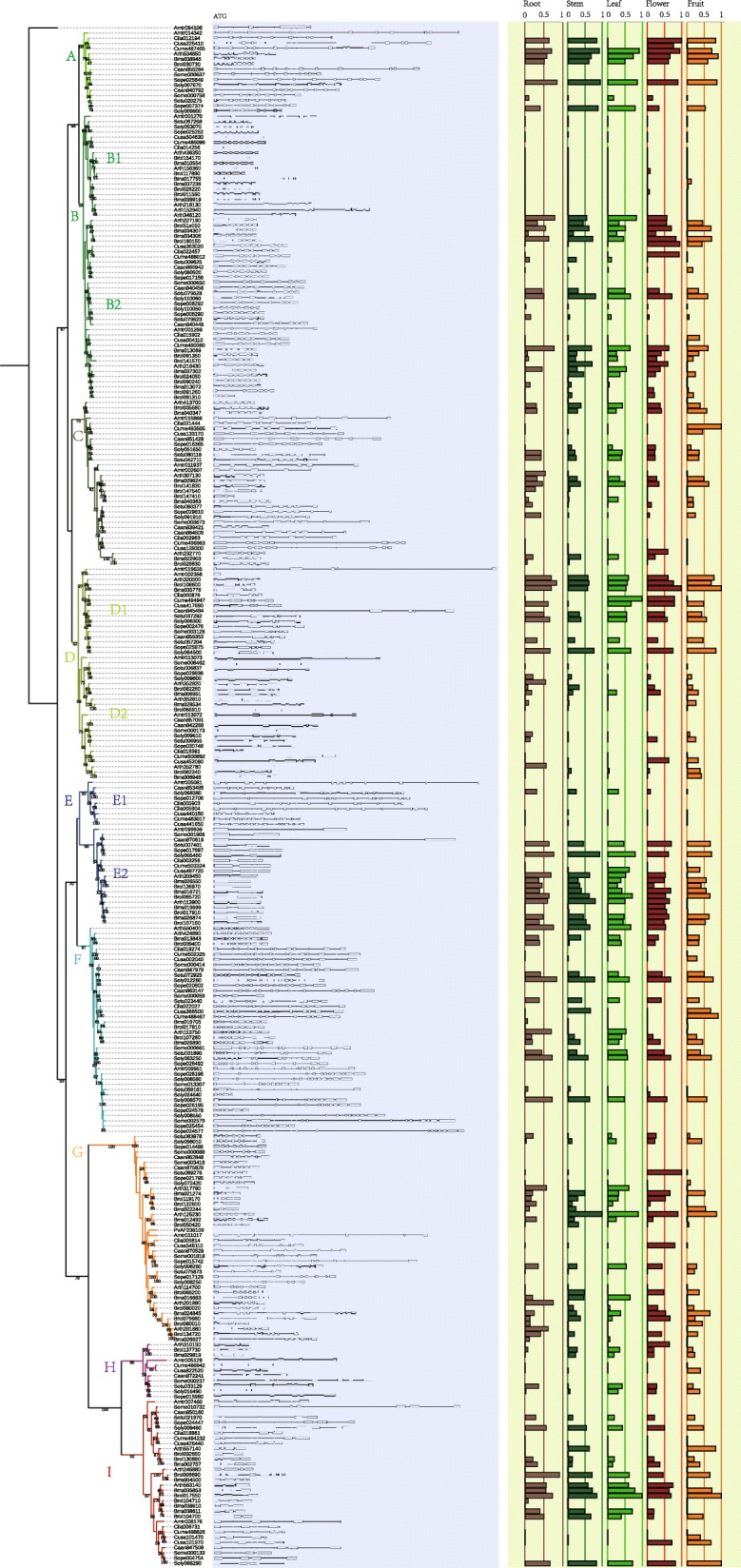
Expression patterns of PAP genes in five tissues. Left: ML tree of 296 PAP amino acid sequences (clades were distinguished by different colors). Middle: intron (line)-exon (box) patterns of PAPs. Length of the longest sequence is 15,316. Displayed by GSDS: http://gsds1.cbi.pku.edu.cn/. Right: Expression levels (0–1 transformed FPKM values) of PAP genes in roots (brown), stems (green), leaves (light green), flowers (red) and fruits (yellow)

A total of 296 PAP sequences (295 from 12 genomes and *Phaseolus vulgaris* AF236109) were used to construct phylogenetic trees using the maximum likelihood (ML) method. Because the Amtr094106 sequence from the basal angiosperm *A. trichopoda* did not form part of any cluster, it was appointed as the hypothetical root. Therefore, the sequences formed nine clades (A to I; Fig. 2). Each clade includes 1−3 sequences from the *A. trichopoda* genome and a certain number of PAPs from Brassicaceae, Solanaceae and Cucurbitaceae (Fig. 3, left). Furthermore, the full-length PAP sequences were analysed by the reciprocal best BLAST hit approach and orthoMCL (see Methods). The clustered ortholog pairs, called ortholog groups, were associated with the clades of ML tree. This indicated an orthologous relationship among clades during evolutionary history.

After extracting gene structural information from the annotation files, we compared the intron-exon patterns of the PAP family, and as expected, members in the same clade shared similar intron-exon patterns. However, between different clades or ortholog groups, the number of exons varied dramatically, ranging from 2 (clade E2) to 14 (clade F). In most clades, regardless of exon distributions, the variation of intron length contributed larger proportion than the exon length to the total length of genes. Take clade E2 and F for example, the diversification of position and length of introns are significant.

Analysis of amino acid sequence revealed slight variation of the five conserved blocks among clades (Fig. 2). The C-terminal three blocks are conserved in all clades, but the two N-terminal blocks are varied in clades H and I, the first block is deleted in clade I, and the metal-ligating residue (Y) in the second block is changed to T and F in clades H and I, respectively. Also, as shown in Fig. 2, all well characterized ~ 35 kDa PAPs are embedded in clade G, while ~ 55 kDa PAPs are in clades A and B. The functional modes of members in the remaining clades still need to be explored.

Differences in selection pressure among clades was detected by one-ratio and two-ratio models using PAML (Table 1). The one-ratio model value of ω (dN/dS) was 0.08, implying a strong negative selection pressure experienced by the PAP family, and different clades have clearly experienced diverse negative or positive pressures. For example, the foreground ω value of clades A and B in two-ratio models indicates more constrained purifying selection compared with the background value obtained by likelihood ratio tests. However, the ω values of clades H and I were estimated to be much greater than 1, and the value for clade H was significantly different from that of the one-ratio model. After testing by the branch-site model, some positive selective sites were identified for clade H, but these positive sites were only significant in Naive Empirical Bayes (NEB) analysis and not Bayes Empirical Bayes (BEB) analysis, the latter of which is recommended by PAML. Positive selection was also insignificant when comparing to the null model, implying that positive selective sites were not truly supported, and may instead reflect relaxed purifying selection. Some complete *Amborella* sequences were retained in clades H and I, suggesting sequences in clades H−I and clades A−G differentiated from each other before the emergence of early angiosperms, then subsequently faced different selection pressures.

**Table 1:** Parameters reflecting changes in selection pressure predicted by models

Lineage-based tests for selection								
model	clade No.	np	lnL	2|ΔlnL|	Significant (*p* < 0.05)	ω	ω (background)	ω (foreground)
one-ratio model		537	−37,685.482872			0.08013		
two-ratio model	A	538	−37,681.586780	7.792184	yes		0.08013	0.00200
	B	538	−37,678.998415	12.968914	yes		0.08020	0.00049
	C	538	−37,683.604012	3.757720	no		0.08030	0.00916
	D	538	−37,685.271690	0.422364	no		0.08011	0.01379
	E	538	−37,684.446480	2.072784	no		0.08031	0.00402
	F	538	−37,684.155074	2.655596	no		0.08034	0.00301
	G	538	−37,685.056969	0.851806	no		0.08018	0.00755
	H	538	−37,682.302812	6.360120	yes		0.08020	inf.
	I	538	−37,683.897444	3.170856	no		0.08019	inf.
Branch-sites test for positive selection of clade H								
model	np	lnL	2|ΔlnL|	Significant	Class 0	Class 1	Class 2a	Class 2b
Null (neutral)	539	−37,663.562931			*p = 0.56355* *ω (background) = 0.08032* *ω (foreground) = 0.08032*	*p = 0.00407* *ω (background) = 1* *ω (foreground) = 1*	*p = 0.42929* *ω (background) = 0.08032* *ω (foreground) = 1*	*p = 0.00310* *ω (background) = 1* *ω (foreground) = 1*
Positive Selection	540	−37,661.849118	3.427626	no (vs Null)	*p = 0.52279* *ω (background) = 0.08042* *ω (foreground) = 0.08042*	*p = 0.00377* *ω (background) = 1* *ω (foreground) = 1*	*p = 0.47005* *ω (background) = 0.08042* *ω (foreground) = inf.*	*p = 0.00339* *ω (background) = 1* *ω (foreground) = inf.*

### Expression patterns of PAP family genes

RNA-seq transcriptomes were downloaded from public databases (Additional file 1: Table S1). Of the 12 genomes, datasets for five tissues (root, stem, leaf, flower, and fruit) were obtained from *A. thaliana*, *B. oleracea*, *B. rapa*, *C. sativus*, *C. melo*, *S. lycopersicum* and *S. tuberosum*. Datasets for Pi starvation treatments (-Pi, +Pi) were obtained from *C. sativus* and *C. lanatus* (Additional file 1: Table S1). In order to increase comparability, normalisation and 0−1 range transformation were performed for each species and dataset (see Methods).

The abundance of PAP transcripts in the five tissues were presented along the tree (Fig. 3, right). Although the data were of different types and were collected from different species, differences in expression levels were largely consistent with differences in sequence similarity. This may reflect the high similarity of regulatory regions. Regarding PAPs expressed in at least one tissue, almost all clades contained some copies that are expressed at high levels, and none of the nine clades are composed entirely of pseudogenes. However, genes in several sub-clades (B1, D2 and E1) do not appear to be expressed in any tissues. It is possible that genes in these clusters lost the ability to be transcribed when regulatory regions became impaired, which presumably occurred later than the formation of the characteristic protein structural features.

Most PAPs were found to be almost equally abundant in all tissues, but some exhibited distinct tissue-specific patterns, with extremely high levels in one of the five tissues. In every clade, sequences from different taxonomic categories displayed more diverse patterns between tissues. For example, in clade B, PAPs of Brassicaceae are more abundant in roots, while Cucurbitaceae PAPs are expressed more abundantly in flowers. In clade D, Brassicaceae PAPs are also expressed preferentially in roots, flowers and fruits, while Cucurbitaceae PAPs are most abundant in leaves and flowers. In clade F, PAPs of Brassicaceae and Solanaceae are expressed maximally in roots, while PAPs of Cucurbitaceae are fruit-specific. Genes in clades C and H are generally present in low abundance except for three genes in *Cucumis* that are expressed highly in fruits.

Transcriptome datasets from *C. sativus* and *C. lanatus* obtained with and without Pi starvation were compared, and three PAPs in *C. sativus* (Csa5M548110, Csa6M366500 and Csa6M504630) and one in *C. lanatus* (Cla012194) were found to be upregulated in the tested tissues (Additional file 1: Table S2). Other PAPs showed no obvious changes.

### Analysis of *cis*-acting elements and annotations reveals potential transcription factors regulating PAP expression

In order to identify putative transcriptional factors based on conserved motifs in promoter regions, sequences 1000 bp upstream from initiation codons of PAPs were analysed. Genes with expression values larger than 0.5 (0−1 transformed, see Methods) in at least one tissue were included in the analysis. Seven sets of upstream sequences were selected as follows: those with maximum expression values in roots (Set 1, Root), stems (Set 2, Stem), leaves (Set 3, Leaf), flowers (Set 4, Flower), fruits (Set 5, Fruit), the sum of all five tissues (Set 1−5, all), and those with upregulated expression values under Pi starvation (Set 6, Pi starvation). Pi starvation-induced PAPs detected in the transcriptomes of *C. sativus* and *C. lanatus*, and some experimentally validated PAPs such as AtPAP10, 12 and 26 in *Arabidopsis*, and SlPAP1 in tomato, were included.

**Fig. 4:**
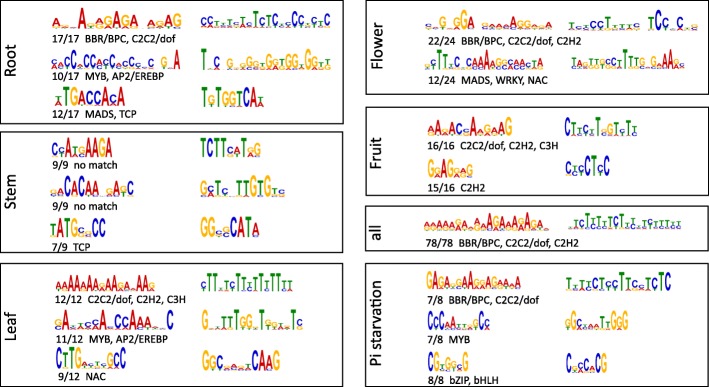
Conserved motifs present in tissue-specific or phosphate starvation-induced PAPs

We then attempted to identify conserved motifs using MEME and upstream sequences in each set, and several conserved motifs in each set were obtained. Motifs were presented if emerged from more than half of all input sequences (Fig. 4). Set 1−5 contains sequences with the lowest degree of expression specificity, and only GAGA repeat motifs were identified. Comparing motifs with published libraries using Tomtom identified BBR-BPC, C2C2-dof and C2H2 as candidate transcription factors. In other sets containing genes specifically expressed in a certain tissue, or under certain environmental conditions (i.e. Pi starvation), similarities and differences in motifs were apparent. As shown in Fig. 4, all sets included the conserved GA-rich motif that was identified in the summed set. However, the ACC/TGG-rich motif was also identified in upstream sequences of PAPs expressed specifically in roots, stems and leaves, implicating MYB- and AP2-EREBP-type transcription factors as candidates. Other motifs identified in tissue subsets identified MADS, TCP, NAC and WRKY as candidate transcription factors. Significant divergence was evident among these motifs. For example, TCP binding sites were identified in the promoters of PAPs expressed in roots and stems, while MADS-type transcription factors appear to be involved in regulating PAPs in roots and flowers. Regarding the Pi starvation subset, the CGTG(G/T)(C/A)G motif was identified, implicating bZIP and bHLH transcription factors as candidates.

## Discussion

### Functional differentiation of extant PAPs was established prior to the emergence of early angiosperms

Organisms have evolved effective mechanisms to maximise external P acquisition and reprioritisation of internal P use to support fundamental biological processes. Because plants are sessile, components facilitating these mechanisms are likely to be highly specialised, and PAPs in vacuoles and rhizospheres are an example. There is a dearth of information on these mechanisms in agriculturally important vegetable species, hence we analysed the structural and evolutionary relationships of PAP family members in vegetable genomes in the present work.

Analysis of variation in copy number did not indicate retention bias among PAPs, suggesting they are largely irreplaceable. Phylogenetic analysis of PAPs identified from 11 higher plants and one early angiosperm revealed the evolutionary path of PAPs. Each of the nine clades includes sequences from the *A. trichopoda* genome and a number of PAPs from Brassicaceae, Solanaceae and Cucurbitaceae, suggesting that PAP diversification had already occurred in the common ancestor of angiosperms.

The PAP family members appear to have undergone negative selection, but the strength of the selective pressure differs between clades (Table 1). Clades A−G experienced negative selection, implying functional conservation of these PAPs, while clades A and B experienced significantly stronger selection pressure. Comparing with previous work, the well-characterised *Arabidopsis* AtPAP10, 12 and 26 enzymes all belong to clades A or B [11, 31], confirming that they are important for Pi starvation-induced acid phosphatase activity. By contrast, clades H and I exhibit more relaxed selective constraints. AtPAP14, 16, 28 and 29 belong to these clades, and changes in active site residues in these clades implies potential functional differences due to altered metal-binding properties. Actually, as the replacement of the tyrosine ligand by either threonine (T) or phenylalanine (F) that would disrupt the charge-transfer transition, they would not strictly be typical purple APs. T and F residues are highly conserved in clades H and I, respectively, and the C-terminal three blocks were particularly conserved. On the other hand, transcription of most members of these clades was normal. Therefore, although no significant positive site was estimated based on the branch-site model, we cannot exclude the possibility that these PAPs are efficient phosphatases. It is worthwhile identifying the explicit functions of PAPs in clade H and I.

PAP fragments with impaired protein-coding regions were not included, and only complete PAPs were analysed to ensure the existent function of enzyme structures. Additionally, transcription of PAPs, another component of function, was also assessed for each clade (Fig. 3). Some clades (C and H) and sub-clades (B1, D2 and E1) were found to absent or transcribed at very low levels, possibly due to recent disruption of regulatory regions.

Together, this evidence suggests that functional diversification of extant PAPs was established prior to the emergence of early angiosperms, and has since been conserved during evolution. However, experimental validation has mainly been reported for PAPs in clades A, B (the ~ 35 kDa form) and G (the ~ 55 kDa form). High level expressions confirmed their functional integrality. As reported in animals, the ~ 35 kDa form PAPs often have redox-active Fe(III)-Fe(II) centers, and the ~ 55 kDa form PAPs often have redox-inactive Fe(III)-Zn(II) or Fe(III)-Mn(II) centers [17]. Maybe the divergence in plants also conforms to this rule. No explicit functions have been confirmed for members of other clades. In addition to acid phosphatase activity, more complex functions are possible. For example, GmPAP3 reportedly participates in salt stress tolerance in soybean [39, 40], and AtPAP25 participates in the defence response against bacteria [41]. Since numerous PAPs have been retained in plant genomes, and because most appear to be transcriptionally active, they may function in P utilisation or other important physiological processes, and further investigation is clearly needed.

### Transcription of PAPs in different tissues and under different environmental conditions is associated with *cis*-acting elements in PAP promoters

PAP genes exhibited tissue-specific expression patterns; some PAPs were almost equally abundant in all tissues, while others were present at extremely high levels in one of the five tissues. This implies that some PAPs may function as typical housekeeping proteins, consistent with roles in P acquisition and utilisation in plants. Others are expressed more flexibly during different growth processes and may help to ensure appropriate nutrient conditions during developmental transitions. Interestingly, PAPs from different taxonomic categories exhibited more diverse patterns, presumably due to evolutionary or developmental reasons. Differences in sampling times and data collection strategies used to generate the different datasets are potential sources of the apparent inconsistencies between genes, and this is likely to be less of an issue for genes with larger differences. Furthermore, given the decent from a common ancestor of extant angiosperms, structural features and regulatory mechanisms should be congenetic. Thus, we concluded that data for genes exhibiting similar tissue-specific expression patterns among taxonomic categories are more likely to be reliable.

Analysis of Pi starvation-induced expression in *C. sativus* and *C. lanatus* failed to identify as many upregulated PAPs as were reported previously in *Arabidopsis* and soybean [21, 23, 30, 31]. This may be due to the shorter treatment duration in experiment that led to the data assessed in the present work, but given the same circumstances, rapid responses usually equate to strong effects. The existing literature claims that a few PAP enzymes exerting strong effects contribute a large proportion of the SAP activity in roots [31, 32]. After checking the upregulation scope of each PAP in previously reported species, we concluded that although a large number of PAPs are upregulated, only a few of the more important enzymes are included in this group. Additionally, those with weak Pi starvation responses are likely to play redundant roles, unlike strongly response PAPs.

Where tissue-specific patterns or Pi starvation-induced patterns were apparent, conserved motifs were identified de novo (Fig. 4). Putative binding sites for transcriptional factors were identified by referring to known libraries, variation between PAPs was evident. By separating PAPs into sets based on specific expression patterns, putative sites for MYB, WRKY, bHLH, C2H2, AP2/EREBP, MADS, TCP, NAC and bZIP transcription factors were identified in the PAP promotor regions. The first four of this list have already been shown to bind upstream of PAP genes [33]. In the summed set (sets 1−5), only the GAGA repeat motif was present in all input sequences. Furthermore, diversification of annotated motifs in each tissue type was evident; TCP was only linked to roots and stems, and MADS to roots and flowers. In addition, bZIP or bHLH binding sites were only present upstream of PAPs induced by Pi starvation, which reflect distinct responses to environmental conditions. Together, these results imply specificity in the promoter regions of genes exhibiting differences in expression.

Although significant from a bioinformatic standpoint, the putative sites are tentative. Because all motifs are comprised of only four bases, conserved motifs can usually bind a variety of transcription factors [42, 43]. Also, if the binding sites of two paired transcription factors overlap, the binding site specificity of an individual factor will be altered [44]. Thus, the accuracy of predicted *cis*-regulatory elements is limited without experimental validation. Nevertheless, de novo identification of conserved motifs can provide insight into the evolutionary history of PAPs with a given expression pattern, and this can be used for investigating the complex regulatory networks of PAPs.

## Conclusion

In this work, we identified genes encoding PAPs in angiosperm genomes including 10 vegetable crops, and performed comprehensive analysis of enzyme structures and transcriptional expression. Phylogenetic analysis of amino acid sequences revealed that functional differentiation of extant PAPs was established prior to the emergence of early angiosperms, and has been conserved among homologs in each clade. Evaluation of transcriptome data and *cis*-acting element prediction based on conserved motifs suggests that PAPs are expressed differently in different tissues or under different environmental conditions due to the presence of *cis*-acting elements in PAP promoters. PAP homologs with complete catalytic domains and appropriate expression profiles can therefore serve as candidates for plant breeding programs or genetic engineering studies.

## Methods

### Identification of PAP homologs

The genomic coding sequence (CDS) of PAPs was downloaded for 12 species from Phytozome (http://www.phytozome.net/), Ensembl Plants (http://plants.ensembl.org/index.html), TAIR (http://www.arabidopsis.org/), BRAD (http://brassicadb/brad/), CuGenDB (http://icugi.org/), Eggplant Genome (http://eggplant.kazusa.or.jp/) and NCBI (https://www.ncbi.nlm.nih.gov/genome/) databases (Additional file 1: Table S1). BLAST [45] and HMMER [46] were used to identify PAP genes based on homology searches. PAP sequences that have been characterised in the literature [14, 21, 22, 25, 39, 47, 48] were used as queries (Additional file 1: Table S3). For BLAST searches, protein sequences of queries were used as inputs in the BLASTp tool, and the resulting hits were filtered by E-value (1e^− 5^). For HMMER searches, query PAPs were first aligned using MAFFT [49], and conserved regions, including the five invariant blocks [14] were used as inputs to build .hmm files with the hmmbuild tool, and .hmm files were used as queries to search target genome peptide sequences with the hmmsearch tool, both of which are in the HMMER package. The threshold E-value was 0.01. Sequences obtained simultaneously by BLASTp and HMMER were extracted for subsequent analysis (Additional file 1: Table S2). In addition, in order to evaluate the number of pseudo-genes, we performed tBLASTn (with a threshold of 1e-5) against the CDS databases. The number of pseudo-genes equals the number of tBLASTn hits minus the number of BLASTp and HMMER hits.

### Phylogenetic analysis of amino acid sequences

Amino acid sequences translated from nucleotide sequences were aligned with MAFFT [49], then transformed into the corresponding codon sequences using PAL2NAL [50]. The best-fit amino acid substitution model WAG+G + I was selected by MEGA [51], and maximum likelihood (ML) analyses were performed using RAxML [52] with 1000 bootstrap replicates.

Ortholog relationships of PAPs were established using the reciprocal best BLAST hit approach [53], with E-values of 1e-30. The identified gene pairs were clustered into ortholog groups by orthoMCL [54]. The inflation parameter was set to 1.5, and all other parameters were set to default values. Ortholog groups were list in Additional file 1: Table S4.

### Selection pressure analysis

To detect changes in evolutionary rate and signatures of positive selection, we analysed the alignments of codon sequences and ML trees under a maximum likelihood framework using the Codeml program in PAML 4.8 [55]. The one-ratio model assumes the same ω (ω = dN/dS, where dN is the non-synonymous substitution rate and dS is the synonymous substitution rate) for all branches. The two-ratio model assumes a foreground ω parameter for each appointed branch and a background ω for all other branches [56]. Models were compared using likelihood ratio tests (LRTs) of the log likelihood (InL), and the 2|ΔlnL| value between models and the degree of freedom were subjected to *chi*-square tests with a significance threshold *p* < 0.05. Since the two-ratio models showed that the ω values for several branches were significantly different from those obtained with the one-ratio models, we used branch-site model A to test for sites that were potentially under positive selection on a given branch. Branch-site model A was compared with the nearly neutral model (M1) [57]. Naive Empirical Bayes analysis and Bayes Empirical Bayes analysis were used to estimate positive sites for foreground lineages.

### Transcriptome analysis

Expression data for different tissues (root, stem, leaf, flower and fruit) and treatments (-Pi, +Pi) were obtained from public databases (Additional file 1: Table S1). RNA-seq read data were first filtered using Perl script IlluQC.pl in the NGS QC toolkit [58], with the paired-end mode and parameters setting as -l 70 and -s 20. Then trimmed reads by TrimmingReads.pl in this toolkit under -l 10. Clean reads then mapped to reference genome sequences by TopHat2 [59], with default settings (−-mate-inner-dist 75 --segment-mismatches 2 --library-type fr-unstranded) for paired-end transcriptomes. FPKM values were calculated and normalised by the Cuffquant and Cuffnorm pipelines in Cufflinks [60].

In order to compare the abundance of transcripts between species, log2-transformed expression values of PAPs were then converted to the 0−1 range within each species using the following formula:

$$ \left(\log 2{\mathrm{FPKM}}_{\mathrm{target}\ \mathrm{PAP}\ \mathrm{in}\ \mathrm{target}\ \mathrm{genome}}-\log 2{\mathrm{FPKM}}_{\mathrm{minimum}\ \mathrm{PAP}\ \mathrm{in}\ \mathrm{target}\ \mathrm{genome}}\right)/\left(\log 2{\mathrm{FPKM}}_{\mathrm{maximum}\ \mathrm{PAP}\ \mathrm{in}\ \mathrm{target}\ \mathrm{genome}}-\log 2{\mathrm{FPKM}}_{\mathrm{minimum}\ \mathrm{PAP}\ \mathrm{in}\ \mathrm{target}\ \mathrm{genome}}\right). $$

Figures were generated by iTol [61], and expression values and gene names are listed in Additional file 1: Table S2.

### Conserved motifs prediction and annotation

Franking sequences of *PAP* genes from 1000 bp upstream of initiation codons were obtained by Ensembl BioMarts [62] or PERL scripts. The MEME suite [63] was used for analysis of conserved motifs among all upstream sequences. First, the MEME program was employed in zoops (zero or one occurrence per sequence) mode to find motifs with a width ranging from 5 to 20 (single nucleotide repeats were ignored). Tomtom was then used to compare predicted motifs with known motifs in published libraries. The ARABIDOPSIS database of DAP motifs [43] was chosen for the target.

## Additional file

Additional file 1: **Table S1.** Information on the genomes and transcriptomes used in this study that were downloaded from public databases. **Table S2.** Sequences used for phylogenetic analysis and expression levels obtained from transcriptome datasets. **Table S3.** Queries used for BLAST searches. **Table S4.** Ortholog groups obtained by orthoMCL. (XLSX 46 kb)

## References

[1] Bieleski RL (1973). Phosphate pools, phosphate transport, and phosphate availability. Ann Rev Plant Physiol.

[2] Hinsinger P (2001). Bioavailability of soil inorganic P in the rhizosphere as affected by root-induced chemical changes: a review. Plant Soil.

[3] Syers JK, Johnson AE, Curtin D (2008). Efficiency of soil and fertilizer phosphorus: reconciling changing concepts of soil phosphorus chemistry with agronomic information.

[4] López-Arredondo DL, Leyva-González MA, González-Morales SI, López-Bucio J, Herrera-Estrella L (2014). Phosphate nutrition: improving low-phosphate tolerance in crops. Annu Rev Plant Biol.

[5] Zhang Z, Liao H, Lucas WJ (2014). Molecular mechanisms underlying phosphate sensing, signaling, and adaptation in plants. J Integr Plant Biol.

[6] Richardson AE, Hocking PJ, Simpson RJ, George TS (2009). Plant mechanisms to optimise access to soil phosphorus. Crop Pasture Sci.

[7] Veneklaas EJ, Lambers H, Bragg J, Finnegan PM, Lovelock CE, Plaxton WC (2012). Opportunities for improving phosphorus-use efficiency in crop plants. New Phytol.

[8] Nannipieri P, Giagnoni L, Landi L, Renella G, Bunemann EK, Oberson A, Frossard E (2011). Role of phosphatase enzymes in soil. Phosphorus in action.

[9] Duff SMG, Sarath G, Plaxton WC (1994). The role of acid phosphatases in plant phosphorus metabolism. Physoil Plantarum.

[10] Veljanovski V, Vanderbeld B, Knowles VL, Snedden WA, Plaxton WC (2006). Biochemical and molecular characterization of AtPAP26, a vacuolar purple acid phosphatase up-regulated in phosphate-deprived *Arabidopsis* suspension cells and seedlings. Plant Physiol.

[11] Tran HT, Qian W, Hurley BA, She YM, Wang D, Plaxton WC (2010). Biochemical and molecular characterization of AtPAP12 and AtPAP26: the predominant purple acid phosphatase isozymes secreted by phosphate-starved *Arabidopsis thaliana*. Plant Cell Environ.

[12] Bozzo GG, Raghothama KG, Plaxton WC (2002). Purification and characterization of two secreted purple acid phosphatase isozymes from phosphate-starved tomato (*Lycopersicon esculentum*) cell cultures. Eur J Biochem.

[13] Bozzo GG, Raghothama KG, Plaxton WC (2004). Structural and kinetic properties of a novel purple acid phosphatase from phosphate-starved tomato (*Lycopersicon esculentum*) cell cultures. Biochem J.

[14] Schenk G, Guddat LW, Ge Y, Carrington LE, Hume DA, Hamilton S (2000). Identification of mammalian-like purple acid phosphatases in a wide range of plants. Gene.

[15] Sträter N, Klabunde T, Tucker P, Witzel H, Krebs B (1995). Crystal structure of a purple acid phosphatase containing a dinuclear Fe(III)-Zn(II) active site. Science.

[16] Guddat LW, McAlpine AS, Hume D, Hamilton S, de Jersey J, Martin JL (1999). Crystal structure of mammalian purple acid phosphatase. Structure.

[17] Schenk G, Mitić N, Hanson GR, Comba P (2013). Purple acid phosphatase: a journey into the function and mechanism of a colorful enzyme. Coord Chem Rev.

[18] Durmus A, Eicken C, Sift BH, Kratel A, Kappl R, Hüttermann J (1999). The active site of purple acid phosphatase from sweet potatoes (*Ipomoea batatas*). Eur J Biochem.

[19] Schenk G, Ge Y, Carrington LE, Wynne CJ, Searle IR, Carroll BJ (1999). Binuclear metal centers in plant purple acid phosphatases: Fe-Mn in sweet potato and Fe-Zn in soybean. Arch Biochem Biophys.

[20] Beck JL, McConachie LA, Summors AC, Arnold WN, De Jersey J, Zerner B (1986). Properties of a purple phosphatase from red kidney bean: a zinc-iron metalloenzyme. Biochim Biophys Acta.

[21] Li D, Zhu H, Liu K, Liu X, Leggewie G, Udvardi M (2002). Purple acid phosphatases of *Arabidopsis thaliana*. J Biol Chem.

[22] Zhang Q, Wang C, Tian J, Li K, Shou H (2011). Identification of rice purple acid phosphatases related to phosphate starvation signalling. Plant Biol.

[23] Li C, Gui S, Yang T, Walk T, Wang X, Liao H (2012). Identification of soybean purple acid phosphatase genes and their expression responses to phosphorus availability and symbiosis. Ann Bot.

[24] González-Muñoz E, Avendaño-Vázquez AO, Montes RAC, de Folter S, Andrés-Hernández L, Abreu-Goodger C (2015). The maize (*Zea mays* ssp. *mays* var. B73) genome encodes 33 members of the purple acid phosphatase family. Front Plant Sci.

[25] Wasaki J, Omura M, Osaki M, Ito H, Matsui H, Shinano T (1999). Structure of a cdna for an acid phosphatase from phosphate-deficient lupin (*Lupinus albus* L.) roots. Soil Sci Plant Nutr.

[26] Bozzo GG, Dunn EL, Plaxton WC (2006). Differential synthesis of phosphate-starvation inducible purple acid phosphatase isozymes in tomato (*Lycopersicon esculentum*) suspension cells and seedlings. Plant Cell Environ.

[27] Zhu H, Qian W, Lu X, Li D, Liu X, Liu K (2005). Expression patterns of purple acid phosphatase genes in *Arabidopsis* organs and functional analysis of *AtPAP23* predominantly transcribed in flower. Plant Mol Biol.

[28] Wang L, Li Z, Qian W, Guo W, Gao X, Huang L (2011). The *Arabidopsis* purple acid phosphatase AtPAP10 is predominantly associated with the root surface and plays an important role in plant tolerance to phosphate limitation. Plant Physiol.

[29] Tadano T, Sakai H (1991). Secretion of acid phosphatase by the roots of several crop species under phosphorus-deficient conditions. Soil Sci Plant Nutr.

[30] Liu PD, Xue YB, Chen ZJ, Liu GD, Tian J (2016). Characterization of purple acid phosphatases involved in extracellular dNTP utilization in *Stylosanthes*. J Exp Bot.

[31] Wang L, Lu S, Zhang Y, Li Z, Du X, Liu D (2014). Comparative genetic analysis of *Arabidopsis* purple acid phosphatases AtPAP10, AtPAP12, and AtPAP26 provides new insights into their roles in plant adaptation to phosphate deprivation. J Integr Plant Biol.

[32] Robinson WD, Park J, Tran HT, Vecchio HAD, Ying S, Zins JL (2012). The secreted purple acid phosphatase isozymes AtPAP12 and AtPAP26 play a pivotal role in extracellular phosphate-scavenging by *Arabidopsis thaliana*. J Exp Bot.

[33] Lin WY, Lin SI, Chiou TJ (2009). Molecular regulators of phosphate homeostasis in plants. J Exp Bot.

[34] Suen PK, Zhang S, Sun SS (2015). Molecular characterization of a tomato purple acid phosphatase during seed germination and seedling growth under phosphate stress. Plant Cell Rep.

[35] Dey N, Sarkar S, Acharya S, Maiti IB (2015). Synthetic promoters *in planta*. Planta.

[36] Cheng F, Wu J, Wang X (2014). Genome triplication drove the diversification of *Brassica* plants. Hortic Res.

[37] Adams KL, Wendel JF (2005). Polyploidy and genome evolution in plants. Curr Opin Plant Biol.

[38] Wang J, Sun P, Li Y, Liu Y, Yang N, Yu J (2017). An overlooked paleotetraploidization in Cucurbitaceae. Mol Biol Evol.

[39] Liao H, Wong F-L, Phang T-H, Cheung M-Y, Li W-YF, Shao G (2003). *GmPAP3*, a novel purple acid phosphatase-like gene in soybean induced by NaCl stress but not phosphorus deficiency. Gene.

[40] Li WYF, Shao G, Lam HM (2008). Ectopic expression of *GmPAP3* alleviates oxidative damage caused by salinity and osmotic stresses. New Phytol.

[41] Ravichandran S, Stone SL, Benkel B, Prithiviraj B (2013). Purple acid phosphatase5 is required for maintaining basal resistance against pseudomonas syringae in arabidopsis. BMC Plant Biol.

[42] Franco-Zorrilla JM, López-Vidriero I, Carrasco JL, Godoy M, Vera P, Solano R (2014). DNA-binding specificities of plant transcription factors and their potential to define target genes. Proc Natl Acad Sci U S A.

[43] O'Malley RC, Huang SC, Song L, Lewsey MG, Bartlett A, Nery JR (2016). Cistrome and epicistrome features shape the regulatory DNA landscape. Cell.

[44] Jolma A, Yin Y, Nitta KR, Dave K, Popov A, Taipale M (2015). DNA-dependent formation of transcription factor pairs alters their binding specificity. Nature.

[45] Altschul SF, Gish W, Miller W, Myers EW, Lipman DJ (1990). Basic local alignment search tool. J Mol Biol.

[46] Eddy SR (2009). A new generation of homology search tools based on probabilistic inference. Genome Informatics.

[47] Hegeman CE, Grabau EA (2001). A novel phytase with sequence similarity to purple acid phosphatases is expressed in cotyledons of germinating soybean seedlings. Plant Physiol.

[48] Miller SS, Liu J, Allan DL, Menzhuber CJ, Fedorova M, Vance CP (2001). Molecular control of acid phosphatase secretion into the rhizosphere of proteoid roots from phosphorus-stressed white lupin. Plant Physiol.

[49] Katoh K, Misawa K, Kuma K-i, Miyata T (2002). MAFFT: a novel method for rapid multiple sequence alignment based on fast Fourier transform. Nucleic Acids Res.

[50] Suyama M, Torrents D, Bork P (2006). PAL2NAL: robust conversion of protein sequence alignments into the corresponding codon alignments. Nucleic Acids Res.

[51] Tamura K, Peterson D, Peterson N, Stecher G, Nei M, Kumar S (2011). MEGA5: molecular evolutionary genetics analysis using maximum likelihood, evolutionary distance, and maximum parsimony methods. Mol Biol Evol.

[52] Stamatakis A, Hoover P, Rougemont J (2008). A rapid bootstrap algorithm for the RAxML web servers. Syst Biol.

[53] Tatusov RL, Koonin EV, Lipman DJ (1997). A genomic perspective on protein families. Science.

[54] Li L, Stoeckert CJ, OrthoMCL RDS (2003). Identification of ortholog groups for eukaryotic genomes. Genome Res.

[55] Yang Z (2007). Paml 4: phylogenetic analysis by maximum likelihood. Mol Biol Evol.

[56] Yang Z (1998). Likelihood ratio tests for detecting positive selection and application to primate lysozyme evolution. Mol Biol Evol.

[57] Yang Z, Nielsen R (2002). Codon-substitution models for detecting molecular adaptation at individual sites along specific lineages. Mol Biol Evol.

[58] Patel RK, Jain M (2012). NGS QC toolkit: a toolkit for quality control of next generation sequencing data. PLoS One.

[59] Trapnell C, Pachter L, Salzberg SL (2009). TopHat: discovering splice junctions with RNA-seq. Bioinformatics.

[60] Trapnell C, Hendrickson DG, Sauvageau M, Goff L, Rinn JL, Pachter L (2013). Differential analysis of gene regulation at transcript resolution with RNA-seq. Nat Biotechnol.

[61] Letunic I, Bork P (2016). Interactive tree of life (iTOL) v3: an online tool for the display and annotation of phylogenetic and other trees. Nucleic Acids Res.

[62] Kinsella RJ, Kahari A, Haider S, Zamora J, Proctor G, Spudich G (2011). Ensembl BioMarts: a hub for data retrieval across taxonomic space. Database.

[63] Bailey TL, Boden M, Buske FA, Frith M, Grant CE, Clementi L (2009). MEME suite: tools for motif discovery and searching. Nucleic Acids Res.

